# Bio-objects and the media: the role of communication in bio-objectification processes

**DOI:** 10.3325/cmj.2013.54.301

**Published:** 2013-06

**Authors:** Pieter Maeseele, Joachim Allgaier, Lucia Martinelli

**Affiliations:** 1 Faculty of Political and Social Sciences, University of Antwerp, Antwerp, Belgium pieter.maeseele@ua.ac.be; 2 Research Center Juelich, Institute of Neurosciences and Medicine, INM-8, Juelich, Germany; 3 Museo delle Scienze, Trento, Italy

## Abstract

The representation of biological innovations in and through communication and media practices is vital for understanding the nature of “bio-objects” and the process we call “bio-objectification.” This paper discusses two ideal-typical analytical approaches based on different underlying communication models, ie, the *traditional* (science- and media-centered) and *media sociological* (a multi-layered process involving various social actors in defining the meanings of scientific and technological developments) approach. In this analysis, the latter is not only found to be the most promising approach for understanding the circulation, (re)production, and (re)configuration of meanings of bio-objects, but also to interpret the relationship between media and science. On the basis of a few selected examples, this paper highlights how media function as a primary arena for the (re)production and (re)configuration of scientific and biomedical information with regards to bio-objects in the public sphere in general, and toward decision-makers, interest groups, and the public in specific.

Modern biotechnology has disrupted the boundaries between what was traditionally understood as “life” and non-living material, eg, by merging objects of human, animal, plant, or synthetic origin. The COST Action *Bio-objects and their boundaries: governing matters at the intersection of society, politics and science* puts forward the concept “bio-object” to get hold of such new entities of life. Its goal is the development of new interdisciplinary analytical tools to improve our understanding of bio-objects and their trajectories from production (conceptual and material development) through application, circulation, and governance in society (1). It approaches bio-objects as temporary contingent products of ongoing bio-objectification processes, which are produced by efforts to tame or control life in a specific locale at a specific time, and as a result bio-objects by definition exceed any characterization as predefined classes of stable entities (2). Characterized by fluidity and mobility, as well as multiple and divergent cultural meanings, these material articulations of new life forms call for analytical tools that allow to track their social life cycles, from creation, over diverse uses, to re-creations, in multiple contexts by multiple social actors. And more specifically, bio-objects also call for a critical examination of how existing (deeply unequal) and contested social relations are mobilized or transformed through these bio-objectification processes.

The aim of this paper is to evaluate how the analytical level of media and communication practices in general, and the analysis of the circulation, (re)production, and (re)configuration of meanings of bio-objects in public and media discourses in specific, could contribute to achieving these goals. Moreover, this analytical level will be argued to open up an important critical arena for observing and registering existing processes of bio-objectification. Advocates of the “biomedia” approach even argue that the products of current biotechnologies can be understood as new forms of media themselves, by merging information and materiality (3).

The representation of certain biological entities in and through communication and media practices is imperative for their identification, stabilization, formulation, definition, framing, etc., ie, for assigning them their nature of “bio-object,” through that process we call “bio-objectification.” When news media pick up a particular frame, this frame contributes to the stabilization of a specific bio-object, and by extension, to the definition about what is life and what is not (eg, about embryonic stem cells). Media function as key vehicles for the (re)production and (re)configuration of scientific and biomedical information, as well as the primary arena in which bio-objects come to the attention of wider society in general, and decision-makers, interest groups, and the public in specific (4). Consequently, media coverage potentially also functions as informal policy advice or even informal governance mechanism, when stakeholders anticipate and respond to the various ways a subject is approached by journalists. On the other hand, media coverage also functions as the primary site for contesting existing institutional definitions of bio-objects and bio-objectification processes, and as a central place for staging transnational resistance to the neoliberal assumptions underlying the European approach to the knowledge-based bio-economy. In short, media are privileged sites for their social revelation, construction, contestation, and criticism.

## Conceptual approaches to media and science

In the literature on media and science, there are two ideal-typical analytical approaches based on different underlying communication models: the traditional approach and media sociological approach (5). We will evaluate both approaches on the extent to which they are able to provide answers to core research questions of the Action, which are also the themes of its respective working groups: (i) How are the boundaries between human and animal, organic and non-organic, living and the non-living opened up and discursively constructed in public and media discourses? (ii) How is the multi-level governance of bio-objects, from the level of the European Union and its Member States to the subpolitical level, and finally in clinics and Laboratories, discursively constructed, contested, and legitimized in public and media discourses? (iii) Which generative (social, economic, political, etc.) relations emerge in these discourses, what are their relations to specific cultural, social, economic, political, and historical frameworks, and what are the implications regarding power, democratic debate, and citizenship?

### The traditional approach

The field of media and science has traditionally been influenced by adjacent fields such as science communication, science popularization, and risk communication. Based on the mutual influence of early communication theory and early sociology of science, the overarching idea has always been and largely continues to be (certainly within the life sciences and policy-making) that scientific knowledge is produced by scientists and experts in a realm separate from the realm of non-scientists to whom it must subsequently be transmitted. The relationship between media and science then is traditionally characterized as a matter – and primarily as a *problem -* of communication and information (6,7), reducing research questions to how well this linear transmission process between the (“unitary” and “consensual”) scientific realm to society functions, either in terms of “adequate” media coverage (ie, studies measuring the level of accuracy) (8) or in terms of “adequate” public understanding (ie, studies measuring the public knowledge of individual scientific “facts”) (9).

This traditional model is science-centered, since it not only makes scientists extraneous to the process and establishes them as hierarchically dominant, but also problematizes media and public, but not science itself. It is also media-centered, since any dislocation in the relationship between science and society is attributed to an inadequate transmission of information. Finally, it regards public acceptance of science and technology as simply a matter of overcoming resistance – from special interest groups, professional mediators (eg, journalists), or citizens – by more and better science diffusion and media coverage, and the promotion of public understanding.

In addition to starting from outdated linear communication models, this model starts from an objectivist concept of science, a positivist view of scientific knowledge, and the assumption that there is always one “correct” interpretation of any techno-scientific controversy; in other words, it starts from an unproblematized notion of scientific consensus. Resulting from a turn in the sociology of science in the 1970s, historians and sociologists from the fields of the sociology of scientific knowledge (SSK) and science and technology studies (STS) have criticized the idea of two separate realms of science and society, questioning (i) the assumption that public discourse only begins where scientific discourse ends (10,11), (ii) the linearity of the communication process (12), and (iii) the neglect of either feedback on core scientific practice or interactivity between different forms of media, scientists, and citizens (13).

Nonetheless, following the publication of a report in 1985 by the Royal Society of London (14), the concept of the *Public Understanding of Science* (PUS) has come to symbolize the tenacity and enduring strength of the traditional model for policy-makers and scientific societies and institutes. Seminal authors in the field such as Durant (15) and Wynne (9) have explained the inflation of the traditional model in the guise of PUS as a response of the scientific establishment to a widely perceived legitimation vacuum and crisis in public trust in a period in which the commercialization of science took off in leading areas of biotechnology. This enduring success of the traditional model in institutional circles is to be found in its ideological and political functions. First, in public discourses it serves as a powerful tool for anyone who derives his or her authority from scientific expertise by providing a repertoire of conceptual and rhetorical devices for foreclosing public, media, or policy debates on bio-objects, such as discourses of “sound science.” Second, since this model only problematizes the media and the public, the dominant PUS agenda continues to naturalize the existing institutionalized culture of science in terms of its representation, organization, patronage, control, and social relations.

### The media sociological approach

A more recent media sociological approach to the relationship between media and science focuses on the meaning-making practices underlying definitions of scientific and technological developments in public and media discourses. Here, the popularization of scientific knowledge is characterized as a multi-layered process in which different social actors (heterogeneous and unequal in power), such as science organizations, industry, consultants, policy-makers, independent scientists, citizen groups, and political and social movements/NGOs are involved in defining the meanings of scientific and technological developments (16). Moreover, media discourses are seen as constituting a site of contestation over science’s representations of science and technology (17). Since science is no longer approached as detached from, but as something culturally defined within, society, science and scientific legitimacy are not assumed to be predefined, but achieved within the communication process itself.

The research questions in this approach focus on understanding (i) how scientific rationality and claims are represented in the media and by whom, (ii) how this relates to issues of access to the media and social debate, and (iii) how these media products are interpreted and used by their various audiences. In other words, the production, representation, and audience reception levels become equally important for analyzing the circulation, re-production and re-configuration of meanings about techno-scientific objects. Furthermore, the mediatization of science itself, ie, professionalization of marketing practices, public relations, and image management within science organizations, also becomes an object of critical concern.

Starting from this approach, many recent empirical studies have revealed a relatively effective control of science’s public image in media discourses, in terms of relatively effective science PR, with journalists often uncritically reproducing PR material (18), a largely affirmative (and in some fields even hyperbolic) character of science reporting (19), a coverage largely reliant on uncontested scientific expertise by drawing from only one institutional source (20), indications of a symbiotic relationship between scientists and their media-contacts, including a high degree of satisfaction among scientists regarding media reporting (18), and, last but not least, cultural resonances that make it harder for critical stories to gain prominence or for critical sources to be accredited with legitimacy (21).

## Role of media and communication in the bio-objectification processes

Now the question arises which of these two approaches is most suitable for the analysis of the circulation, (re)production, and (re)configuration of meanings of bio-objects, which are characterized, not as predefined classes of stable entities, but as fluid, mobile, and imbued with multiple and divergent cultural meanings. A vital difference between both approaches is their radically diverging interpretation of the relationship between media and science, in which we find an intricate process of in- and exclusion, of defining the boundaries, of “science” in “science communication” or “science in the media” (5). The traditional model is concerned with strategies to safeguard institutionalized conceptions of techno-scientific objects by distinguishing these from “false” manifestations instead of “alternative” ones, starting from an assumption of communication practices as the (in)efficient communication of predefined matters. The media sociological approach refers to strategies to reveal the meaning-making processes underlying definitions of techno-scientific objects, starting from an assumption of communication practices as indefinite articulations of meaning.

While both interpretations represent a struggle between two politico-ideological models for interpreting the relationship between media and science, only the latter opens the discursive space for approaching public and media discourse as a site of cultural struggle, while the aim of the former is exactly to delegitimize the acknowledgment of a cultural struggle. The traditional approach then is inadequate to study bio-objectification processes, since it cannot account for competing or contingent discursive constructions of bio-objects or diverse social actors who are often in the process of re-configuring institutional definitions. To the contrary, by starting from an unproblematized notion of scientific consensus (which just needs to be communicated efficiently), it starts from an instrumental role of media in promoting science and the acceptance of institutionally predefined definitions of bio-objects. By problematizing only public perceptions and attitudes as well as communication and media practices, it is impossible to contest and criticize the nature, origin, and implications of specific bio-objectification processes.

Therefore, we argue for a reflexive and sociologically informed approach to study how media and communication practices could improve our understanding of bio-objectification processes in society. A first important question that must be asked when thinking about bio-objects and bio-objectification processes in the media is “who actually has a voice and is represented as spokesperson for bio-objects?” Conrad (22) found in news stories about genetics that people affected by certain biomedical conditions only rarely have a voice in the media (that is, only when they are organized): generally, the “bioscientific experts” are found speaking about new biological objects and topics. However, also among these experts there are differences in terms of media appearances: it is not necessarily their scientific expertise that counts, but also their characteristics as journalistic sources, for instance if they are willing to return journalists’ phone calls in time.

The three core research questions of the Action (the definition of the boundaries between human and non-human or living non-living matter, the governance of bio-objects, and bio-objectification, and the emergence of social and economic relations) all involve important bioethical questions and concerns. Research by Kruvand (23) demonstrated that concerning bioethical issues in the media, one expert (Arthur L. Caplan) managed to become the *de facto* bioethics representative for about two decades; a powerful position for shaping media and public discourses on bioethical issues. The reason why this particular academic could become so powerful is that he is particularly media-savvy and has an excellent understanding of how the media operate.

In addition to identifying who has voice, it is imperative to also have a better understanding of the communication processes involved, when we want to increase our understanding of how bio-objects are identified, defined, represented, and communicated in various societal contexts. Furthermore, we also need to take new information and communication infrastructures, such as the internet and smart phones, into account if we want to understand current bio-objectification processes (24). Much has changed since the Bodmer Report was published in 1985. Before this new communication era, people affected by certain biomedical conditions were generally denied access to the media, and more importantly, to biomedical research *on their conditions*. Today, patients have been found to start using the extraordinary mobilizing power of social media and the internet to advocate their concerns. For instance, the hypothesized cause of multiple sclerosis, a condition called chronic cerebrospinal venous insufficiency, led a researcher to propose a new treatment named “the liberation procedure.” In most countries this hypothesis received almost no attention. In Canada, however, hundreds of Facebook groups, pages, and events with thousands of participants got together to influence research priorities and “the liberation procedure” became an issue of national interest. What the clinicians and researchers took from this episode was that traditional approaches for communicating scientific findings to the public are insufficient today and that clinicians need to find new ways of interacting with citizens and patients (25). But even more than that has happened in the meantime. For instance, some scientists encourage citizens to participate in their research via the internet: in the popular online game “Foldit,” interested citizens take part in folding proteins in virtual models, and particular promising variants will then be synthesized in the laboratory (26). Various actors also suppose that recent developments in the media and communication infrastructure are about to revolutionize everyday medical practice, particularly concerning the data and biological information about patients. Here it is assumed that the patients will soon be empowered by media developments to take all their personal biological data in their own hands, which is a precondition for a truly “personalized medicine” (27). Obviously, this development will bring new challenges in terms of the bio-virtual and the ethics surrounding the issue of bio**-**data. However, online networks and platforms like PatientsLikeMe.com are already allowing patients to link up and share their information and experiences. In this context, Akst (28) reports on terminally ill patients abandoning the medical system altogether and starting to experiment on themselves by sharing the outcomes of experimental treatments among each other on the web.

Cases such as this one pose challenging questions in terms of bio-identities, bio-objects, and bio-data. To get a hold on complex bio-social issues such as these it is crucial to not only take the role of media and communication practices and technological communication infrastructures into account, but also the most suitable approach, especially in the light of tremendous democratic challenges with regards to science in society.
